# SProtP: A Web Server to Recognize Those Short-Lived Proteins Based on Sequence-Derived Features in Human Cells

**DOI:** 10.1371/journal.pone.0027836

**Published:** 2011-11-16

**Authors:** Xiaofeng Song, Tao Zhou, Hao Jia, Xuejiang Guo, Xiaobai Zhang, Ping Han, Jiahao Sha

**Affiliations:** 1 Department of Biomedical Engineering, Nanjing University of Aeronautics and Astronautics, Nanjing, China; 2 State Key Laboratory of Reproductive Medicine, Department of Histology and Embryology, Nanjing Medical University, Nanjing, China; 3 Department of Gynecology and Obstetrics, The First Affiliated Hospital with Nanjing Medical University, Nanjing, China; International Centre for Genetic Engineering and Biotechnology, Italy

## Abstract

Protein turnover metabolism plays important roles in cell cycle progression, signal transduction, and differentiation. Those proteins with short half-lives are involved in various regulatory processes. To better understand the regulation of cell process, it is important to study the key sequence-derived factors affecting short-lived protein degradation. Until now, most of protein half-lives are still unknown due to the difficulties of traditional experimental methods in measuring protein half-lives in human cells. To investigate the molecular determinants that affect short-lived proteins, a computational method was proposed in this work to recognize short-lived proteins based on sequence-derived features in human cells. In this study, we have systematically analyzed many features that perhaps correlated with short-lived protein degradation. It is found that a large fraction of proteins with signal peptides and transmembrane regions in human cells are of short half-lives. We have constructed an SVM-based classifier to recognize short-lived proteins, due to the fact that short-lived proteins play pivotal roles in the control of various cellular processes. By employing the SVM model on human dataset, we achieved 80.8% average sensitivity and 79.8% average specificity, respectively, on ten testing dataset (TE1-TE10). We also obtained 89.9%, 99% and 83.9% of average accuracy on an independent validation datasets iTE1, iTE2 and iTE3 respectively. The approach proposed in this paper provides a valuable alternative for recognizing the short-lived proteins in human cells, and is more accurate than the traditional N-end rule. Furthermore, the web server SProtP (http://reprod.njmu.edu.cn/sprotp) has been developed and is freely available for users.

## Introduction

Proteins are the chief actors within the cell. All proteins in mammal cells are continually being degraded and replaced. Some cytosolic enzymes have half-lives as short as 10 minutes, whereas others last for days. The fluctuations in their expression are fundamental for metabolism, cell cycle control and communication between a cell and environment. The cell's proteolytic machinery must be highly selective and tightly regulated, since the accelerated destruction of an essential protein or the failure to degrade a short-lived regulatory protein could drastically alter cell function [1].

The continual destruction of cell proteins may appear to be wasteful, but it serves several important homeostatic functions [2]. The rapid removal of rate-limiting enzymes and regulatory proteins is essential for the control of growth and metabolism. For example, the progression of cells through the mitotic cycle requires the programmed destruction of the critical regulatory proteins called cyclins. The rapid degradation of specific proteins permits an adaptation to new physiologic conditions. And the tumor suppressor protein p53 is a short-lived protein that is maintained at low, often undetectable, levels in normal cells [3]. If p53 was stabilized in response to an activating signal, such as DNA damage, its expression level will rise rapidly and inhibit cell growth.

The study of factors affecting those short-lived proteins has begun in the last century. The amino acid sequence composition was shown to be closely related to protein half-lives. In 1986, Rogers et al. proposed the PEST hypothesis that proteins with PEST sequence tend to undergo rapid intracellular degradation [4]. In the same year, Bachmair et al. proposed the N-end rule that certain terminal amino acid yielded proteins with very short half-lives whereas others rendered the protein very stable [5] [14]. Other protein sequence motifs such as D-box and KEN box were also found to be important for protein stability regulation [6]–[8].

Most of above studies for protein degradation were previously performed on only a few or individual proteins due to the technical difficulty in global profiling. While in 2006, Archana et al. performed profiling of over 3,000 protein stability in yeast [9]. Follow-up bioinformatics analysis of this data showed that among all the physical and sequence features of proteins considered, the most significant feature that correlates with intracellular degradation is disorder region in protein 3-D structure [10], but the correlation is very weak.

Short-lived proteins are usually regulatory proteins. Most of the studies for short-lived proteins have been performed on individual protein in human. And there is still lack of the characteristics profile analysis for these short-lived proteins. During our study, Huang, et al. published a paper in which they employed Nearest Neighbor Algorithm to identify four-type half-life protein based on some features [11]. But they used a two-step classification strategy to predict the four types with the accuracy of about seventy percent for each step. The overall accuracy for prediction of each type is relatively poor. In this paper, we will mainly investigate in depth protein sequence and structural features to reveal some strong factors related to those short-lived proteins in human cells. And a web server was also constructed to predict short-lived proteins in human with high accuracy.

## Materials and Methods

### Relevant Dataset construction

The datasets of human protein half-lives used in this paper were downloaded from literature in Science [12] resulting from high-throughput experiment for proteome-scale protein-turnover within mammalian cells. We manually checked the sequences of ORFs encoding proteins, and removed genes with multiple ORFs, which can not have unambiguous protein stability information mapped in Sherry et al's data. And we obtained 5,818 different proteins with unambiguous stability data, and employed them in our analysis. In order to evaluate our method correctly, we filtered the dataset with two ways. Firstly, we used BLAST to remove the redundant proteins using 20% sequence identity as the cutoff. Using such low threshold is to ensure a good prediction generalization [13]. Because proteins secreted into extracellular space can significantly decrease the proteins content in the cell, only the intracellular protein degradation was investigated in this paper, while the secreted proteins as annotated in Uniprot database [14] were removed. After filtering, we obtained 4838 high qualified full-length human proteins. The filtered 980 (5818–4838) sequences are denoted as “FIL_dataset”. According to the definition of PSI (Protein Stability Index), we found that there are 510 proteins belonged to the short half-lived (PSI<2.0, being equivalent to 30 minutes).

The training dataset was obtained by randomly selecting 80% of the positive samples and the same number of negative samples in order to maintain data balance. Thus, the training dataset contains 408 positive and 408 negative samples, and the remaining proteins including 102 positive and 3920 negative samples were employed as the testing dataset. To better assess the variance of the estimation, this procedure was repeated 10 times resulting in 10 training datasets (denoted as TR1-TR10) and 10 corresponding testing datasets (denoted as TE1-TE10), respectively.

The first independent validation dataset (denoted as iTE1) were downloaded from literature [15] in which the protocol “dynamic SILAC” was employed to profile the intracellular stability of about 600 proteins from human A549 adenocarcinoma cells. The second independent validation dataset (denoted as iTE2) were downloaded from literature [16] in which the new proposed protocol “bleach-chase” in 2011 was employed to profile the intracellular stability of about 100 proteins from human non–small cell lung cancer cell line. The third independent validation dataset (denoted as iTE3) were downloaded from literature [17]–[18] in which the parallel metabolic pulse labelling was employed in mammalian cells. Because many protein sequences were not uniquely determined by mass spectrometry, and not possible to have their sequence features accurately calculated, we considered only proteins with unique protein sequence determined from the dataset. Thus, we constructed the third independent validation dataset including 1573 long-lived proteins and only one short-lived protein in iTE3. Due to the limitation of their methods, they mentioned that it was not possible to quantify half lives of short-lived proteins (<30 min) accurately [17]–[18]. Therefore, most of the proteins in this dataset are long-lived proteins.

### Feature extraction

We have examined a number of features based on protein sequences and structures that are possibly relevant to the classification of short-lived proteins. Some features are involved because they are known to be relevant to short-lived proteins, while others are taken into account because of their statistical relevance to our classification problem.

As we have known, the short-lived protein stability in a cell can be somewhat determined by its structure and sequence information, and the amino acid sequence is the basis to investigate the protein function. Therefore, we analyzed the amino acid components and distribution of the protein sequence. Firstly we statistically analyzed single peptide and di-peptide composition yielding a feature vector of 420 dimensions. Due to the polypeptide sequences variation in evolutionary process, analyzing the composition of classified amino acids will be more reasonable [19]–[21]. We divided the 20 amino acids into 6 classes according to physiochemical properties: class a (I, V, L, M), class b (F, Y, W), class c (H, K, R), class d (D, E), class e (Q, N, T, P) and class f (A, C, G, S). We then analyzed the composition (C) of each class, and their combined dyad and triplet. Along with the descriptor of composition (C), the transition (T) and distribution (D) were also used to describe the global composition of amino acids classes, in which T denotes the relative frequency of transfers from one class to another along the protein sequence and D denotes the chain length within which the first, 25%, 50%, 75% and 100% of the amino acids of a particular class are located, respectively [19]–[21]. The results of the literature [19]–[21] indicated that the features of composition (C), the transition (T) and distribution (D) are closely related to protein function. Thus 303 feature elements are employed to represent the three descriptors of classified amino acids: 258 for C (6 for each class, 36 for dyad and 216 for triplet), 15 for T and 30 for D. We also take into account some general features such as the protein length, hydrophobic value, sulfur content, isoelectric point, and N-end amino acid.

Structure-related factors are also important for the degradation of short-lived proteins. In this work, we considered the protein secondary structure components such as alpha-helix, beta-strand and coil contents, which were predicted by PSIPRED [22].

Other classical degradation motifs such as short sequence motifs (destruction box and Ken box) are signals for a more specific degradation mechanism that serves primarily to regulate the function of proteins involved in the cell cycle. Destruction-box content was estimated by the “Destruction Box Motif (D-box) Finder” algorithm proposed by Dana Reichmann and his co-workers, which is available at http://bioinfo2.weizmann.ac.il/~danag/d-box/form.html. The D-box identification is implemented by comparing the query sequence with blocks representing the D-box motifs from cyclin A, cyclin B, securin, and geminin protein families. We took the existence of these four kinds of blocks in the protein as a set of features to describe the stability of short-lived proteins. The KEN box was determined by a sequence search for the KENxxxN/D motif. Therefore, we obtained 5 features representing degradation motifs.

Moreover, we took into consideration about the low complexity region (LCR) as an important feature related to short-lived protein. The LCR is a protein region consisting of a very small variety of residues. We examined the numbers of LCR, the length of maximum LCR and the total length of LCR in every sequence by the program of “SEG” [23] in order to investigate the implication with the stability of short-lived proteins.

The N-terminal residue has been expected to be significantly correlated with the protein half-life [24], [25]. The N-end rule indicates a close relationship between a protein half-life and its N-terminal residue, which can be roughly summarized as: destabilizing residues mainly include F, L, W, Y, I, R, K, H, D, E, C, N and Q, while stabilizing residues are mainly M, P, A, S, T, G and V. Thus, we analyzed the N-terminal residue of short-lived and long-lived protein sequences after retrieving the UniProt database. A feature vector of 20 dimensions was used to represent the occurrence of 20 amino acids at the N-terminal position.

PEST regions are generally accepted to be associated with short-lived proteins [26]. The features we extracted from protein sequences using “epestfind” method [22] include the number of potential PEST, the length of maximum potential PEST, the score of maximum PEST region, and the occurrence position of each PEST region in a sequence.

Disordered region in a protein is another important signal for short-lived protein degradation. Some degradation motifs such as D-box tend to fall into the locally disordered regions [27]. Moreover, disordered proteins are more widespread in eukaryotic proteomes. Therefore, 4 disorder-related features extracted by IUPred [28] were taken into consideration including the number of disordered regions, the total length of disordered regions, the length of maximum disordered region, and the average score of disordered regions.

In this study, signal peptides and transmembrane regions are found to be highly related to protein stability, thus we took the existence of signal peptides, the existence of transmembrane regions and the total length of transmembrane regions in a protein as three features in the initial feature list.

Post-translational modification (PTM), which is the chemical modification of a protein after its translation, is closely correlated with the protein function. Thus we investigated possible relationships between kinds of PTMs and protein degradation. In general, PTMs includes phosphorylation, glycosylation, N-acetylation, ubiquitination, and so on. In this study, features focusing on protein phosphorylation and glycosylation were considered including the number of phosphorylation site in serine and threonine respectively, the number of C-glycosylation site, N-glycosylation site and G-glycosylation site.

In the end, we totally obtained 776 features as displayed in Table 1(details see Table S2), which can be roughly grouped into four categories: (1) amino acid composition, (2) physicochemical properties, (3) structure-related characteristics, and (4) degradation-related motifs.

**Table 1:** Features derived from protein sequence.

	feature terms	feature for dataset	abbreviation
amino acids	amino acids content	mono-peptide(20)	AA_*
Content		di-peptide(400)	
(723)	grouped amino acids content	single(6)	aa_*
		dyad (36)	
		triplet(216)	
		transition(15)	*_>*
		distribution(30)	num%*
physicochemical	sequence length	sequence length(1)	len.
property (4)	isoelectric point	isoelectric point(1)	isoele.
	sulphur content	sulphur content(1)	Sulphur
	hydrophobicity of protein	hydrophobicity of protein(1)	Hydrophobicity
structure- related	disorder region	the total length of disorder regions(1)	disorder_len
(7)		the average of scores(1)	disorder_score
		the number of disorder regions(1)	disorder_num
		length of max disorder region(1)	disorder_max
	protein secondary structure	helix content(1)	helix
		sheet content(1)	sheet
		turn content(1)	turn
degradation motif	KEN box	the existence of KEN box(1)	KEN
(35)	destruction box	geminin content(1)	D_g
		cyclinA content(1)	D_cA
		cyclinB content(1)	D_cB
		securin content(1)	D_s
	PEST region	number of PEST regions	PEST_num
		max length of PEST regions	PEST_max
		the average of PEST scores	PEST_score
		the relative position of PEST regions	PEST_posi
	low complexity region	total length of LCRs(1)	LCR_len.
		the number of LCRs (1)	LCR_num
		length of max LCRs (1)	LCR_max
	N terminal	animo acids of N end(20)	N_*
	the existence of signal peptide	the existence of signal peptide(1)	SP
	transmembrane	transmembrane enrichment(1)	TM
		transmembrane region length(1)	TM_len.
Protein	phosphorylation	the content of modification site(3)	Phos
Modification (7)	C-glycosylation	the content of modification site(1)	Cglyc
	N-glycosylation	the content of modification site(1)	Nglyc
	O-glycosylation	the content of modification site(2)	Oglyc
totally 776			

### Feature evaluation and classification

We employed F-value [29] as a feature selection criterion to assess the actual discriminating performance of each feature on the recognition of short-lived proteins, which can be calculated as:
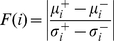
(1)where 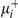 and 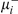 denote the mean values of the ith feature on positive and negative samples, respectively, while 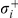 and 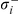 denote the standard deviations of the ith feature on positive and negative samples, respectively.

We defined the propensity score of feature in short-lived or long-lived proteins as follows,
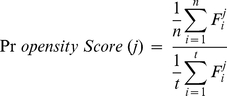
(2)


In which 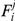 is represented as the value of 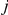
th feature in the 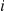
th protein; 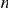 is represented as the number of short-lived proteins or long-lived proteins; 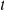 is represented as the number of all proteins. All 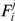 should be scaled to the range 0–1 before using formula (2). Thus the propensity score of >1 indicated that the feature is tend to be higher enriched either in short-lived proteins or in long-lived proteins.

To screen out discriminative features and remove the irrelevant ones, we first removed the redundant features based on Pearson correlation coefficient. We found that 160 pairs of attributes are strongly and positively correlated ranging 0.50–0.99 with P<0.001 in the human training datasets. Therefore, we removed attributes with lower F-values among these correlated features, and obtained 616 features to construct the SVM model. Then we ranked the initial features based on their F-values and selected the ones with F-value not less than 0.1. After that, we applied analysis of variance (ANOVA) to evaluate their discriminative power, and further removed the features with p-value more than 0.001. Finally, we obtained the 211∼254 distinctive features in 10 human training dataset (TR1-TR10) respectively to construct the SVM calssifier [30], [31] (see Table S3). As expected, SVM classifiers with selected features show consistent and comparable classification accuracies on human datasets.

Therefore, we established 10 SVM-based classifier models with 10 human training datasets (TR1-TR10) to distinguish the positive(short-lived proteins) from the negative training data (long-lived proteins), using the program of libsvm-2.88. with RBF kernel function.

To obtain the best model, there are two parameters c and g to be optimized. Using the automatic parameter selection script grid.py supplied by LIBSVM [32], the optimized parameters c and g in each classifier can be seen in Table S1.

## Results and Discussion

### Predicting short-lived proteins with different cutoff

**Figure 1:**
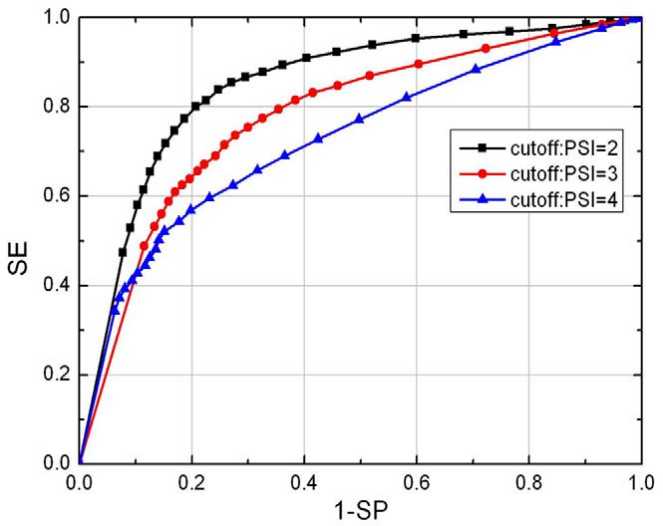
ROC curves of the proposed SProtP using different cutoff.

By using the feature selection method described in [30], criterion of F-value proposed by Golub et al. [29], and redundancy removal, 211∼254 non-redundant features calculated from protein sequences were left for training and prediction. Using all the filtered features components, we evaluated the performance of three different classifiers: “short”-“medium/long/extra-long” classifier, “short/medium”-“long/extra-long” classifier, and “short/medium/long”-“extra-long” SVM-based classifier by ten-fold cross-validation and on corresponding testing dataset. The comparison of three AUC areas showed that “short”-“medium/long/extra-long” classifier performs best (see Fig. 1).

Protein degradation is a regulated process, and it may involve recognition of certain motif or degradation signal in short-lived proteins [33]. Thus, short-lived proteins can be better predicted from features derived from protein protein sequences. It is known that some short-lived proteins have important regulatory roles such as p53 [3]. Thus we analyzed classifier to predict short-lived proteins in advance.

### Prediction performance of short-lived proteins

Employing the selected features which would have strong relevance to protein degradation (see Table S3), we established the 10 classification model based on SVM with 10 human training datasets (TR1-TR10). Each model was obtained by optimizing model parameters to achieve the best ten-fold cross-validation performance on each training dataset, and then tested on corresponding testing dataset. The prediction performances of the 10 classifiers on 10 human testing datasets (TE1-TE10) are generally consistent, ranging from 77.5% to 83.3% for the sensitivity and from 78.8% to 81.2% for specificity, which are provided in detail in Table S1. We also applied 10 classifier models in human filetered dataset (FIL_dataset), we achieved average sensitivity of 81.5% and specificity of 71.1% respectively. The AUC value of prediction results on human filtered datasets (FIL_dataset) using human model is 0.819, which indicated that the classifier proposed in this study generalized very well.

### Analysis of reduced optimal feature set

**Figure 2:**
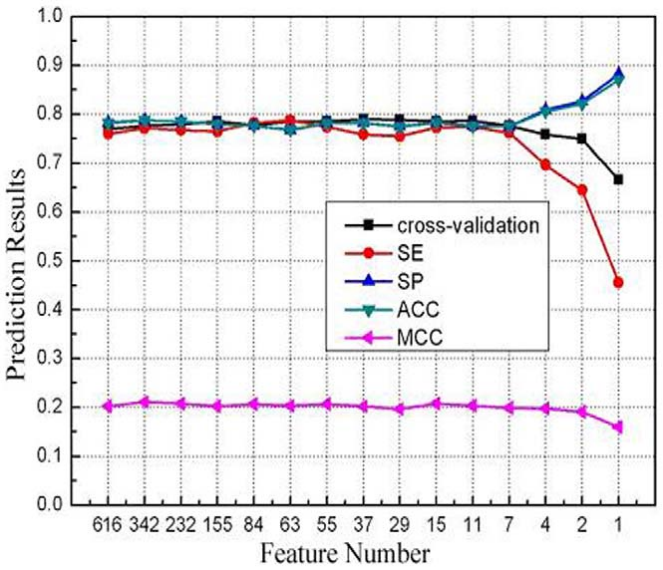
The AUC value of SProtP model varies as feature number in human dataset.

To obtain an optimal set of features, we further reduced the number of features for the “short”-“medium/long/extra-long” classifier, and evaluated the prediction performance of each classifier. We found that the number of features can be further reduced from 616 to 11 without decrease the performance (see Fig. 2). Using the 11 feature components the classifer can obtain an AUC of 0.831.

**Figure 3:**
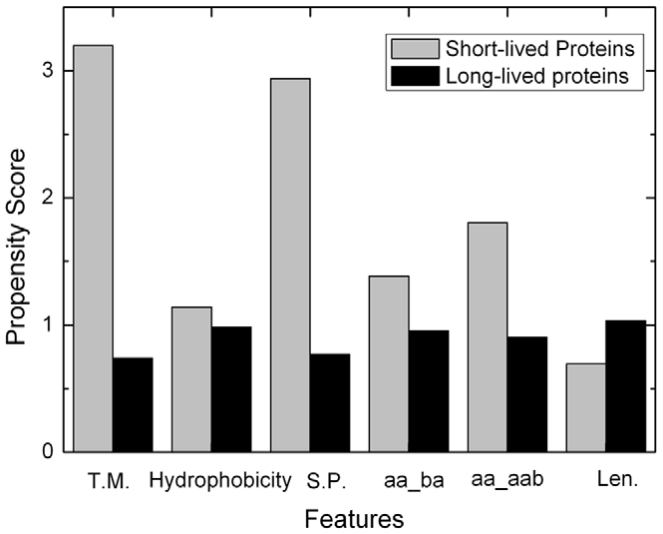
The propensity score of each features for short-lived and long-lived proteins.

Analysis of the 11 feature components showed four classes of features: hydrophobic features (LL - two continuous hydrophobic aromatic amino acid, ba/aab/bb – two or three continuous amino acids with one hydrophobic aromatic amino acid and one or two hydrophobic aliphatic amino acids, TM - transmembrane region, and Hydrophobicity), signal peptide, protein length, and an acidic amino acid or short sequence motif containing an charged amino acid (see Table 2). The propensity score of 6 distinct features for short-lived and long-lived proteins were shown in Fig. 3. It has already been known that protein length affects the half life [10], [12]. Thus, in our analysis, we normalized all the quantitative features against protein sequence length.

**Table 2:** Reduced optimal 11 feature set information.

No.	features	Feature description	F-value
1	TM	transmembrane enrichment	0.4653
2	Hydrophobicity	hydrophobicity of protein	0.4162
3	SP	Signal peptide	0.4114
4	aa_ba	composition of amino acid class b and a combined dyad	0.2950
5	aa_aab	composition of amino acid class a, a, and b combined triplet	0.2875
6	len.	sequence length	0.2827
7	aa_da	composition of amino acid class d and a combined dyad	0.2722
8	AA_D	composition of amino acid “ aspartic acid”	0.2682
9	aa_cd	composition of amino acid class c and d combined dyad	0.2430
10	AA_LL	composition of amino acid “leucine”	0.2408
11	aa_bb	composition of amino acid class b and b combined dyad	0.2255

**Figure 4:**
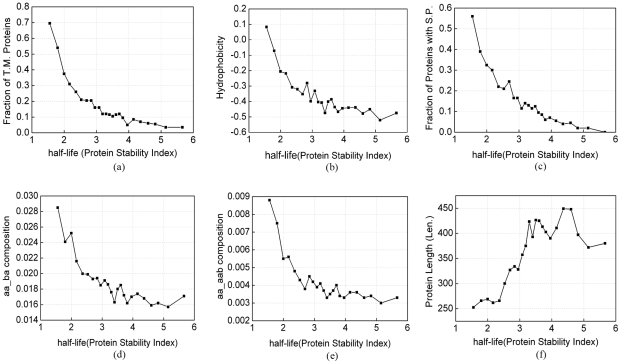
Features varies as protein half-lives.

It is interesting that signal peptide can affect protein half life (see Fig. 3 and Fig. 4). Of the total 510 proteins having shorter half-lives with PSI less than 2, 229 proteins (45%) contain signal peptides. However, of the total 1288 proteins having longer half lives with PSI greater than 4, only 37 proteins (3%) have signal peptides. SignalP predicts signal peptides cleaved by signal peptidase I [34]. Proteins with such signal peptide can be secreted to the extracellular space, and they can also directed to specific organelles in the cell if they have other specific signals, e.g. the protein will be retended in the ER if it holds an “ER retention signal” [34]. We assigned the proteins to subcellular localizations using annotations from UniProt database [35]. Fisher's exact test showed that the proteins with short half lives are significantly enriched in extracellular proteins (p value <0.0001). These proteins are secreted immediately after synthesis, they are expected to have shorter intracellular half lives. In addtion, we found the proteins with short half lives are also significantly enriched in ER proteins (p value = 1.49E-6).

Hydrophobic features are strongly enriched in short-lived proteins (see Fig. 3 and Fig. 4). In contrast, proteins with hydrophilic characters, such as positively-charged amino acids (glutamic acid and aspartic acid), have reversed distributions.

It is possible that hydrophobic interactions are an important mechanism for regulated degradation. It has been known that hydrophobic character of the tyrosine in IκBα is important for its rapid turnover [36]. And addition of hydrophobic carboxy-terminal residues to smRS-GFP protein could dramatically reduce protein levels in the haloarchaeon Haloferax volcanii possibly through proteasome pathway. [37].

Transmembrane proteins can be degraded by specific pathways. In our localization analysis, we also found significant enrichment of fast degrading proteins in cytoplasmic membrane (Fisher's exact test: p = 1.78E-14). Cytoplasmic membrane is not in a static state, accompanied with cyclic generation and membrane movement. Endocytosis is the degradative rout to lysosomes followed by extracellular material and also by certain plasma membrane proteins which will not be recycled to the plasma membrane for re-usage [38]. The endosomal sorting complex required for transport-I (ESCRT-I) complex, which is conserved from yeast to humans, directs the lysosomal degradation of ubiquitinated transmembrane proteins [39]. And an E3 ligases, RSP5, has been found to ubiquinate membrane proteins, and regulate their turnover [40], [41]. The continuous degradation of membrane proteins by lysosome may be the cause of fast turnover of cytoplasmic membrane proteins. This may also cause the phenomenon that transmembrane proteins tend to undergo fast turnover because many transmembrane proteins are located on cytoplasmic membrane.

### SProtP evaluation

In addition, an independent datasets (iTE1, iTE2 and iTE3) as introduced in Method section was used to reevaluate the effectiveness of the proposed SProtP in this paper. Because global mass spectrometry–based proteomics [15], the new proposed “bleach-chase” [16], and parallel metabolic pulse labeling [17]–[18] in mammalian cells were not suitable for detecting low abundance and rapidly turned over proteins [42], these datasets (iTE1, iTE2 and iTE3) mainly contains stable proteins. Owning to the deletion and replacement of some protein accession numbers in the most recent version of UniProt database, 526 negative samples were finally used as the independent dataset iTE1. We obtained 89.9% of average accuracy using the SVM model constructed in human Hela cells. There are about 100 long-lived proteins as negative samples in iTE2 [16], We even obtained 99% of average accuracy when implementing the SProtP model in iTE2. The iTE3 included 1573 long-lived proteins and only one short-lived protein [17]–[18], we obtained 83.9% of average accuracy on iTE3 using our proposed SProtP. These results indicated that the SProtP model proposed in this paper has good generalization, and can recognize those short-lived proteins and long-lived proteins accurately.

ROC curve of the proposed SProtP was also plotted in Fig. 1. The AUC value of prediction results on human testing datasets using human model is 0.848, which is acceptable. The above results are summarized in Table 3.

**Table 3:** Comparison between proposed SProtP and N-end rule.

	SProtP	N-rule
5-fold cross-validation	0.81±1.65	—
TP	82±2	55±2
FN	20±2	47±2
TN	3126±37	2236±10
FP	794±37	1684±10
SE	0.808±0.018	0.539±0.019
SP	0.798±0.009	0.570±0.003
ACC	0.798±0.009	0.570±0.002
MCC	0.231±0.009	0.035±0.006
AUC	0.848±0.013	—

Till now, the N-end rule has been studied in many proteins sequences and it is employed to predict protein half-lives by the ProtParam tool (http://www.expasy.ch/tools/protparam.html). To compare our approach with ProtParam tool, we predicted short-lived proteins in human testing datasets (TE1-TE10). The results showed that ProtParam achieved an overall prediction accuracy 57.0% in human (see Table 3). Thus, our approach outperforms the N-end rule based method ProtParam.

It should be noted that the prediction performance can be improved if there is accurate measure of protein half-lives. Sherry et al's data are subject to noise inherent in the technology used [43]. The microarray was used to measure protein half-lives, but it is already known that microarray is subject to noise [44].

### The web server construction

Based on the “short”-“medium/long/extra-long” classifier using 11 feature components from protein sequence, we developed software Short-lived Protein Prediction in Human (SProtP Human). And it was made freely accessable academically via http://reprod.njmu.edu.cn/sprotp. SProtP Human doesn't require any data other from sequence. Huang et al incorporated seven characters to achieve less than 70% accuracy. These seven characters are (1) KEGG enrichment scores of the protein and its neighbors in network, (2) subcellular locations, (3) polarity, (4) amino acids composition, (5) hydrophobicity, (6) secondary structure propensity, and (7) the number of protein complexes the protein involved. Some of the characters such as KEGG enrichment scores and the number of protein complexes the protein involved are difficult to accurately calculate for less studied proteins. But SProtP Human does not involve this problem, and is easier to be implemented. It can achieve an accuracy of 79.8%. It will provide a rich resource for biologists the study of protein turnover, evaluation of the efficiency of RNAi on protein level.

### Recognizing short-lived proteins

With the availability of the human protein half-life data, we found that sequence-derived features of protein could globally influence their half-lives in mammalian cells. The approach presented in this paper provide a valuable alternative for recognizing short-lived proteins at proteome scale in human cells. However, its accuracy may be affected by many factors such as cell types or stress conditions. It should be noted that protein degradation is very complex in eukaryotic cells. For example, E3 ubiquitin protein ligase family, which provide selectivity to the proteasome-ubiquitin degradation pathway, may number in hundreds in humans [45]. They function under a controlled spatial and temporal regulation. Moreover, some proteins may have variable half-lives in different cell types under different conditions. More experimental studies are required for better understanding protein degradation regulation.

### Conclusion

By investigating the sequence-based features related to protein degradation, a new approach was developed to recognize the short-lived proteins in human cells which are important for cell cycle progression, signal transduction, and differentiation. The average sensitivity and specificity on 10 human testing dataset were 80.8% and 79.8%, respectively. We also obtained 89.9%, 99% and 83.9% of accuracy in an independent datasets iTE1, iTE2 and iTE3, respectively.

As a conventional prediction method for protein half-lives, N-end rule has been employed by researchers for a long time [46] for its simpleness. However, the N-end rule-based methods are far from satisfactory, the new approach proposed in this paper provides a more accurate predictor for short-lived proteins in mammalian cells, and it is provided as a free online tool, SProtP Human, at http://reprod.njmu.edu.cn/sprotp.

## Supporting Information

Table S1 **Evaluation on 10 human testing datasets using corresponding training model.**
(PDF)

Table S2 **Feature List.**
(PDF)

Table S3 **Feature selection in 10 human training dataset.**
(PDF)
