# Supplementary material for: SProtP: A Web Server to Recognize Those Short-Lived Proteins Based on Sequence-Derived Features in Human Cells
Source: PLoS One. 2011 Nov 16;6(11):e27836. doi: 10.1371/journal.pone.0027836 (PMC3218052; doi:10.1371/journal.pone.0027836)
Supplement: Table S1 — Evaluation on 10 human testing datasets using corresponding training model. (PDF) [file pone.0027836.s001.pdf]

Table S1 Evaluation on 10 human testing datasets using corresponding training model

Dataset	c	g	rate(%)	TP	FN	TN	FP	SE	SP	ACC	MCC	AUC
TE1	2	0.031250	78.92	81	21	3088	832	0.794	0.788	0.788	0.218	0.842
TE2	2	0.007813	82.97	85	17	3134	786	0.833	0.799	0.800	0.242	0.854
TE3	0.5	0.031250	81.50	83	19	3088	832	0.814	0.788	0.788	0.226	0.832
TE4	0.5	0.031250	79.78	82	20	3133	787	0.804	0.799	0.799	0.230	0.836
TE5	2	0.031250	78.92	81	21	3088	832	0.794	0.788	0.788	0.218	0.842
TE6	2	0.007813	80.64	83	19	3184	736	0.814	0.812	0.812	0.244	0.869
TE7	2	0.007813	79.53	82	20	3155	765	0.804	0.805	0.805	0.235	0.850
TE8	2	0.007813	82.97	85	17	3134	786	0.833	0.799	0.800	0.242	0.854
TE9	0.5	0.031250	81.50	83	19	3088	832	0.814	0.788	0.788	0.226	0.832
TE10	2	0.007813	82.97	79	23	3172	748	0.775	0.809	0.808	0.227	0.865
mean	—	—	80.97	82	20	3126	794	0.808	0.798	0.798	0.231	0.848
std.	—	—	1.65	2	2	37	37	0.018	0.009	0.009	0.009	0.013
