# Supplementary material for: SProtP: A Web Server to Recognize Those Short-Lived Proteins Based on Sequence-Derived Features in Human Cells
Source: PLoS One. 2011 Nov 16;6(11):e27836. doi: 10.1371/journal.pone.0027836 (PMC3218052; doi:10.1371/journal.pone.0027836)
Supplement: Table S2 — Feature List. (PDF) [file pone.0027836.s002.pdf]

Table S2 Feature List

index	features	index	features
1-20	mono-peptide	726	number of phosphorylation site in Threonine
21-420	di-peptide	727	the number of phosphorylation site in Tyrosine
421-426	sigle grouped aa	728	the number of C-glycosylation site
427-462	diplet grouped aa	729	the number of N-glycosylation site
463-678	triplet grouped aa	730	O-glycosylation site (Serine)
679	sequence length	731	O-glycosylation site (Threonine).
680	Sulphur content	732	distribution of grouped aa. (a) in 0% position of sequence
681	Hydrophobicity	733	distribution of grouped aa. (b) in 0% position of sequence
682	isoelectric point	734	distribution of grouped aa. (c) in 0% position of sequence
683	total length of disorder regions	735	distribution of grouped aa. (d) in 0% position of sequence
684	the average of disorder scores	736	distribution of grouped aa. (e) in 0% position of sequence
685	the number of disorder regions	737	distribution of grouped aa. (f) in 0% position of sequence
686	max length of disorder regions	738	distribution of grouped aa. (a) in 25% position of sequence
687	helix content	739	distribution of grouped aa. (b) in 25% position of sequence
688	sheet content	740	distribution of grouped aa. (c) in 25% position of sequence
689	coil content	741	distribution of grouped aa. (d) in 25% position of sequence
690	the existence of KEN box	742	distribution of grouped aa. (e) in 25% position of sequence
691	geminin content	743	distribution of grouped aa. (f) in 25% position of sequence
692	cyclinA content	744	distribution of grouped aa. (a) in 50% position of sequence
693	cyclinB content	745	distribution of grouped aa. (b) in5 0% position of sequence
694	securin content	746	distribution of grouped aa. (c) in 50% position of sequence
695	total length of low complexity region	747	distribution of grouped aa. (d) in 50% position of sequence
696	the number of low complexity region	748	distribution of grouped aa. (e) in 50% position of sequence
697	length of max low complexity region	749	distribution of grouped aa. (f) in 50% position of sequence
698	existence of signal peptide	750	distribution of grouped aa. (a) in 75% position of sequence
699	number of PEST regions	751	distribution of grouped aa. (b) in 75% position of sequence
700	max length of PEST regions	752	distribution of grouped aa. (c) in 75% position of sequence
701	the average of PEST scores	753	distribution of grouped aa. (d) in75% position of sequence
702	the relative position of PEST regions	754	distribution of grouped aa. (e) in 75% position of sequence
703	N terminal (amino acid F)	755	distribution of grouped aa. (f) in 100% position of sequence
704	N terminal (amino acid L)	756	distribution of grouped aa. (a) in 100% position of sequence
705	N terminal (amino acid W)	757	distribution of grouped aa. (b) in 100% position of sequence
706	N terminal (amino acid Y)	758	distribution of grouped aa. (c) in 100% position of sequence
707	N terminal (amino acid I)	759	distribution of grouped aa. (d) in 100% position of sequence
708	N terminal (amino acid R)	760	distribution of grouped aa. (e) in 100% position of sequence
709	N terminal (amino acid K)	761	distribution of grouped aa. (f) in 100% position of sequence
710	N terminal (amino acid H)	762	transition of grouped aa. (a-b)
711	N terminal (amino acid D)	763	transition of grouped aa. (a-c)
712	N terminal (amino acid E)	764	transition of grouped aa. (a-d)
713	N terminal (amino acid C)	765	transition of grouped aa. (a-e)
714	N terminal (amino acid N)	766	transition of grouped aa. (a-f)
715	N terminal (amino acid Q)	767	transition of grouped aa. (b-c)
716	N terminal (amino acid M)	768	transition of grouped aa. (b-d)
717	N terminal (amino acid P)	769	transition of grouped aa. (b-e)
718	N terminal (amino acid A)	770	transition of grouped aa. (b-f)
719	N terminal (amino acid S)	771	transition of grouped aa. (c-d)
720	N terminal (amino acid T)	772	transition of grouped aa. (c-e)
721	N terminal (amino acid G)	773	transition of grouped aa. (c-f)
722	N terminal (amino acid V)	774	transition of grouped aa. (d-e)
723	transmembrane enrichment	775	transition of grouped aa. (d-f)
724	transmembrane region length	776	transition of grouped aa. (e-f)
725	number of phosphorylation site in Serine		
