# Supplementary material for: SProtP: A Web Server to Recognize Those Short-Lived Proteins Based on Sequence-Derived Features in Human Cells
Source: PLoS One. 2011 Nov 16;6(11):e27836. doi: 10.1371/journal.pone.0027836 (PMC3218052; doi:10.1371/journal.pone.0027836)
Supplement: Table S3 — Feature selection in 10 human training dataset. (PDF) [file pone.0027836.s003.pdf]

Table S3 Feature selection in 10 human training dataset

TR1		TR2		TR3		TR4		TR5		TR6		TR7		TR8		TR9		TR10	
index	Fvalue	index	Fvalue	index	Fvalue	index	Fvalue	index	Fvalue	index	Fvalue	index	Fvalue	index	Fvalue	index	Fvalue	index	Fvalue
723	0.508	723	0.556	723	0.551	723	0.515	723	0.508	723	0.527	723	0.469	723	0.556	723	0.551	723	0.529
681	0.441	681	0.492	681	0.451	681	0.427	681	0.441	681	0.429	681	0.434	681	0.492	681	0.451	724	0.424
724	0.424	724	0.484	724	0.430	424	0.393	724	0.424	724	0.407	424	0.390	724	0.484	724	0.430	681	0.417
698	0.389	463	0.435	424	0.390	698	0.390	698	0.389	424	0.386	698	0.363	463	0.435	424	0.390	698	0.409
424	0.377	424	0.412	698	0.372	724	0.370	424	0.377	698	0.379	421	0.349	424	0.412	698	0.372	463	0.393
464	0.368	698	0.402	464	0.367	463	0.369	464	0.368	463	0.358	493	0.342	698	0.402	464	0.367	424	0.383
428	0.340	427	0.400	427	0.350	421	0.349	428	0.340	493	0.344	463	0.341	427	0.400	427	0.350	427	0.350
2	0.333	464	0.393	433	0.349	493	0.341	2	0.333	421	0.340	724	0.337	464	0.393	433	0.349	493	0.342
679	0.331	421	0.375	463	0.347	427	0.339	679	0.331	427	0.336	2	0.331	421	0.375	463	0.347	2	0.334
463	0.330	2	0.369	2	0.347	2	0.328	463	0.330	464	0.333	427	0.329	2	0.369	2	0.347	468	0.331
427	0.329	428	0.366	422	0.342	679	0.326	427	0.329	2	0.323	679	0.324	428	0.366	422	0.342	421	0.330
421	0.327	468	0.353	421	0.334	468	0.295	421	0.327	679	0.319	428	0.323	468	0.353	421	0.334	428	0.321
499	0.318	433	0.351	679	0.334	432	0.282	499	0.318	428	0.316	464	0.318	433	0.351	679	0.334	464	0.309
445	0.303	422	0.343	499	0.329	1	0.281	445	0.303	469	0.309	422	0.302	422	0.343	499	0.329	433	0.306
469	0.296	493	0.342	428	0.326	499	0.277	469	0.296	445	0.306	1	0.298	493	0.342	428	0.326	679	0.302
433	0.290	469	0.341	493	0.317	7	0.274	433	0.290	433	0.299	7	0.297	469	0.341	493	0.317	422	0.297
422	0.285	445	0.340	468	0.317	428	0.269	422	0.285	1	0.296	433	0.283	445	0.340	468	0.317	499	0.297
684	0.284	499	0.316	469	0.314	457	0.263	684	0.284	643	0.295	460	0.279	499	0.316	469	0.314	445	0.296
494	0.278	679	0.314	445	0.305	460	0.259	494	0.278	468	0.292	684	0.273	679	0.314	445	0.305	643	0.280
493	0.277	3	0.310	505	0.304	450	0.256	493	0.277	432	0.285	468	0.272	3	0.310	505	0.304	469	0.274
1	0.276	460	0.306	434	0.297	433	0.254	1	0.276	499	0.284	469	0.272	460	0.306	434	0.297	1	0.272
457	0.268	643	0.305	442	0.288	494	0.253	457	0.268	457	0.278	448	0.271	643	0.305	442	0.288	457	0.270
460	0.260	432	0.296	457	0.286	464	0.252	460	0.260	422	0.275	442	0.267	432	0.296	457	0.286	432	0.257
685	0.258	1	0.295	643	0.285	445	0.252	685	0.258	7	0.270	3	0.266	1	0.295	643	0.285	484	0.254
442	0.256	457	0.289	432	0.276	448	0.250	442	0.256	442	0.265	499	0.262	457	0.289	432	0.276	494	0.254
643	0.254	423	0.283	1	0.274	422	0.250	643	0.254	505	0.254	457	0.261	423	0.283	1	0.274	448	0.251
468	0.249	529	0.276	19	0.272	643	0.249	468	0.249	684	0.254	643	0.251	529	0.276	19	0.272	589	0.248
432	0.243	147	0.272	3	0.271	680	0.242	432	0.243	143	0.243	147	0.250	147	0.272	3	0.271	450	0.239
3	0.242	442	0.269	448	0.270	470	0.241	3	0.242	470	0.242	450	0.249	442	0.269	448	0.270	7	0.239
553	0.240	505	0.268	423	0.265	147	0.238	553	0.240	147	0.239	494	0.246	505	0.268	423	0.265	423	0.233
448	0.237	504	0.264	553	0.260	42	0.228	448	0.237	728	0.237	725	0.246	504	0.264	553	0.260	728	0.232
505	0.236	434	0.260	460	0.255	505	0.225	505	0.236	494	0.235	42	0.242	434	0.260	460	0.255	458	0.230
423	0.236	7	0.259	494	0.254	146	0.225	423	0.236	448	0.235	432	0.241	7	0.259	494	0.254	460	0.230
644	0.230	684	0.259	42	0.250	423	0.222	644	0.230	434	0.230	685	0.241	684	0.259	42	0.250	3	0.229
42	0.229	447	0.255	470	0.249	19	0.220	42	0.229	553	0.229	683	0.239	447	0.255	470	0.249	454	0.229
589	0.228	143	0.251	589	0.249	684	0.218	589	0.228	3	0.228	680	0.236	143	0.251	589	0.249	434	0.227
680	0.226	494	0.249	458	0.244	454	0.214	680	0.226	460	0.228	434	0.233	494	0.249	458	0.244	664	0.225
683	0.220	589	0.245	7	0.239	484	0.213	683	0.220	685	0.226	447	0.232	589	0.245	7	0.239	505	0.223
484	0.219	685	0.244	504	0.239	589	0.211	484	0.219	5	0.225	143	0.231	685	0.244	504	0.239	5	0.222
19	0.218	458	0.240	143	0.237	458	0.211	19	0.218	649	0.225	589	0.231	458	0.240	143	0.237	147	0.222
447	0.215	146	0.238	728	0.237	442	0.210	447	0.215	423	0.223	470	0.230	146	0.238	728	0.237	680	0.218
143	0.212	450	0.235	147	0.236	434	0.209	143	0.212	484	0.223	505	0.229	450	0.235	147	0.236	684	0.218
147	0.211	725	0.234	450	0.232	725	0.209	147	0.211	589	0.222	529	0.226	725	0.234	450	0.232	19	0.217
7	0.211	448	0.233	5	0.231	683	0.208	7	0.211	146	0.222	19	0.225	448	0.233	5	0.231	470	0.216
382	0.210	683	0.230	447	0.231	3	0.207	382	0.210	42	0.219	728	0.221	683	0.230	447	0.231	42	0.212
454	0.210	19	0.229	683	0.230	469	0.204	454	0.210	680	0.219	445	0.221	19	0.229	683	0.230	447	0.211
450	0.210	484	0.227	382	0.228	644	0.204	450	0.210	454	0.215	458	0.220	484	0.227	382	0.228	474	0.211
529	0.208	42	0.222	684	0.228	447	0.201	529	0.208	450	0.213	5	0.217	42	0.222	684	0.228	146	0.211
573	0.203	553	0.220	146	0.227	701	0.198	573	0.203	689	0.208	423	0.215	553	0.220	146	0.227	430	0.208

725	0.199	500	0.219	701	0.223	702	0.195	725	0.199	529	0.206	484	0.212	500	0.219	701	0.223	442	0.207
470	0.198	649	0.219	59	0.223	685	0.192	470	0.198	67	0.206	644	0.208	649	0.219	59	0.223	649	0.207
474	0.196	67	0.218	500	0.219	453	0.192	474	0.196	458	0.202	449	0.207	67	0.218	500	0.219	529	0.205
449	0.195	430	0.217	484	0.217	5	0.192	449	0.195	447	0.201	689	0.206	430	0.217	484	0.217	143	0.201
434	0.193	644	0.215	644	0.215	127	0.191	434	0.193	63	0.198	664	0.205	644	0.215	644	0.215	701	0.198
699	0.193	664	0.214	454	0.210	689	0.189	699	0.193	683	0.197	699	0.204	664	0.214	454	0.210	583	0.198
15	0.192	470	0.211	685	0.209	143	0.187	15	0.192	430	0.195	594	0.203	470	0.211	685	0.209	438	0.198
702	0.191	661	0.210	689	0.208	529	0.186	702	0.191	644	0.194	701	0.196	661	0.210	689	0.208	683	0.193
700	0.190	59	0.208	6	0.207	562	0.185	700	0.190	571	0.193	382	0.196	59	0.208	6	0.207	347	0.192
146	0.190	167	0.207	67	0.207	728	0.185	146	0.190	648	0.193	41	0.193	167	0.207	67	0.207	685	0.191
701	0.190	530	0.207	576	0.207	487	0.183	701	0.190	438	0.191	649	0.191	530	0.207	576	0.207	644	0.190
145	0.189	474	0.205	664	0.207	649	0.182	145	0.189	504	0.189	454	0.191	474	0.205	664	0.207	41	0.188
696	0.189	438	0.204	699	0.204	430	0.181	696	0.189	382	0.188	146	0.189	438	0.204	699	0.204	725	0.188
430	0.188	583	0.204	478	0.204	443	0.179	430	0.188	725	0.188	438	0.187	583	0.204	478	0.204	68	0.185
458	0.187	487	0.201	700	0.203	699	0.179	458	0.187	687	0.188	437	0.186	487	0.201	700	0.203	504	0.184
59	0.186	63	0.200	725	0.201	583	0.178	59	0.186	474	0.183	122	0.185	63	0.200	725	0.201	625	0.183
67	0.186	227	0.199	529	0.199	437	0.178	67	0.186	19	0.183	583	0.185	227	0.199	529	0.199	594	0.182
6	0.185	437	0.196	14	0.197	553	0.175	6	0.185	449	0.182	59	0.182	437	0.196	14	0.197	15	0.181
649	0.183	728	0.196	583	0.197	59	0.175	649	0.183	500	0.180	67	0.179	728	0.196	583	0.197	127	0.180
504	0.182	443	0.196	680	0.196	604	0.174	504	0.182	701	0.179	553	0.178	443	0.196	680	0.196	530	0.180
438	0.180	163	0.194	437	0.192	504	0.173	438	0.180	664	0.178	36	0.177	163	0.194	437	0.192	154	0.178
627	0.179	5	0.193	702	0.191	15	0.173	627	0.179	530	0.177	473	0.175	5	0.193	702	0.191	506	0.177
68	0.177	680	0.192	650	0.189	4	0.171	68	0.177	347	0.172	604	0.173	680	0.192	650	0.189	157	0.176
648	0.177	454	0.191	63	0.186	594	0.171	648	0.177	401	0.172	68	0.173	454	0.191	63	0.186	553	0.175
500	0.176	576	0.188	159	0.185	347	0.167	500	0.176	487	0.170	167	0.173	576	0.188	159	0.185	22	0.173
728	0.175	382	0.187	591	0.183	661	0.167	728	0.175	466	0.170	474	0.173	382	0.187	591	0.183	122	0.172
167	0.174	459	0.187	28	0.183	63	0.166	167	0.174	699	0.170	702	0.170	459	0.187	28	0.183	500	0.170
5	0.174	489	0.186	573	0.182	523	0.166	5	0.174	661	0.169	663	0.170	489	0.186	573	0.182	59	0.170
437	0.173	402	0.185	443	0.181	382	0.166	437	0.173	163	0.169	700	0.168	402	0.185	443	0.181	689	0.169
11	0.172	347	0.184	488	0.180	449	0.166	11	0.172	700	0.167	687	0.168	347	0.184	488	0.180	67	0.168
496	0.171	466	0.184	696	0.179	486	0.164	496	0.171	68	0.167	696	0.167	466	0.184	696	0.179	574	0.168
583	0.170	6	0.181	474	0.172	700	0.164	583	0.170	122	0.165	11	0.166	6	0.181	474	0.172	523	0.166
47	0.167	488	0.181	587	0.171	11	0.163	47	0.167	573	0.165	157	0.166	488	0.181	587	0.171	573	0.166
689	0.166	68	0.180	163	0.171	56	0.162	689	0.166	696	0.164	22	0.165	68	0.180	163	0.171	699	0.166
141	0.166	127	0.180	347	0.170	67	0.161	141	0.166	583	0.164	402	0.165	127	0.180	347	0.170	167	0.164
107	0.166	663	0.178	438	0.170	335	0.160	107	0.166	28	0.162	347	0.164	663	0.178	438	0.170	202	0.164
127	0.165	449	0.176	649	0.170	157	0.159	127	0.165	328	0.161	487	0.163	449	0.176	649	0.170	151	0.163
478	0.165	8	0.176	401	0.169	36	0.159	478	0.165	594	0.161	504	0.161	8	0.176	401	0.169	700	0.163
663	0.165	47	0.176	648	0.169	473	0.158	663	0.165	123	0.160	478	0.160	47	0.176	648	0.169	163	0.163
133	0.162	573	0.176	487	0.167	650	0.157	133	0.162	726	0.159	127	0.158	573	0.176	487	0.167	182	0.163
594	0.161	523	0.176	594	0.166	268	0.155	594	0.161	673	0.157	107	0.158	523	0.176	594	0.166	449	0.163
123	0.161	640	0.176	133	0.165	573	0.155	123	0.161	598	0.157	467	0.157	640	0.176	133	0.165	14	0.162
487	0.161	4	0.176	687	0.165	37	0.153	487	0.161	4	0.156	591	0.157	4	0.176	687	0.165	496	0.162
182	0.161	46	0.176	41	0.164	592	0.152	182	0.161	402	0.156	63	0.157	46	0.176	41	0.164	702	0.161
473	0.159	478	0.175	157	0.163	319	0.151	473	0.159	523	0.156	322	0.156	478	0.175	157	0.163	36	0.160
163	0.159	701	0.175	510	0.163	687	0.151	163	0.159	46	0.155	430	0.156	701	0.175	510	0.163	478	0.158
22	0.158	141	0.175	601	0.162	87	0.149	22	0.158	41	0.155	182	0.156	141	0.175	601	0.162	459	0.156
524	0.158	453	0.173	15	0.162	598	0.149	524	0.158	574	0.153	341	0.156	453	0.173	15	0.162	439	0.156
347	0.158	496	0.173	66	0.161	500	0.148	347	0.158	22	0.152	466	0.155	496	0.173	66	0.161	123	0.155
63	0.156	157	0.170	439	0.161	41	0.148	63	0.156	167	0.152	453	0.155	157	0.170	439	0.161	437	0.154
608	0.154	20	0.170	22	0.159	202	0.147	608	0.154	478	0.151	506	0.155	20	0.170	22	0.159	650	0.154
574	0.154	15	0.169	429	0.159	113	0.146	574	0.154	608	0.151	4	0.155	15	0.169	429	0.159	6	0.153
601	0.153	689	0.169	122	0.158	648	0.146	601	0.153	562	0.151	496	0.152	689	0.169	122	0.158	11	0.153

159	0.152	395	0.167	506	0.158	474	0.145	159	0.152	47	0.150	26	0.152	395	0.167	506	0.158	4	0.152
661	0.152	575	0.166	430	0.158	478	0.144	661	0.152	59	0.149	601	0.150	575	0.166	430	0.158	601	0.150
441	0.152	14	0.166	123	0.157	26	0.144	441	0.152	127	0.149	556	0.150	14	0.166	123	0.157	166	0.149
530	0.151	594	0.165	673	0.157	266	0.144	530	0.151	640	0.148	573	0.149	594	0.165	673	0.157	604	0.149
486	0.149	401	0.164	661	0.156	166	0.143	486	0.149	26	0.147	523	0.149	401	0.164	661	0.156	27	0.149
400	0.149	571	0.164	390	0.154	663	0.142	400	0.149	443	0.147	598	0.148	571	0.164	390	0.154	266	0.149
453	0.148	182	0.163	473	0.154	601	0.141	453	0.148	591	0.147	686	0.147	182	0.163	473	0.154	443	0.148
466	0.148	27	0.162	530	0.153	466	0.141	466	0.148	506	0.145	628	0.147	27	0.162	530	0.153	382	0.147
697	0.148	574	0.162	466	0.153	6	0.141	697	0.148	702	0.145	488	0.147	574	0.162	466	0.153	401	0.144
488	0.148	699	0.162	4	0.153	530	0.141	488	0.148	149	0.144	640	0.146	699	0.162	4	0.153	648	0.143
8	0.148	673	0.161	127	0.153	321	0.140	8	0.148	658	0.144	524	0.144	673	0.161	127	0.153	488	0.143
571	0.146	328	0.160	453	0.152	68	0.138	571	0.146	27	0.144	510	0.144	328	0.160	453	0.152	46	0.142
48	0.146	28	0.159	663	0.152	390	0.138	48	0.146	11	0.143	697	0.144	28	0.159	663	0.152	487	0.142
664	0.146	40	0.159	628	0.152	640	0.138	664	0.146	37	0.142	319	0.143	40	0.159	628	0.152	453	0.142
576	0.146	700	0.158	287	0.150	141	0.133	576	0.146	106	0.142	592	0.143	700	0.158	287	0.150	489	0.140
27	0.146	650	0.157	182	0.149	664	0.133	27	0.146	168	0.142	145	0.142	650	0.157	182	0.149	63	0.140
55	0.146	598	0.156	302	0.149	123	0.133	55	0.146	453	0.141	15	0.142	598	0.156	302	0.149	608	0.140
686	0.145	266	0.156	571	0.148	673	0.132	686	0.145	157	0.141	648	0.141	266	0.156	571	0.148	490	0.139
625	0.145	128	0.156	145	0.148	65	0.132	625	0.145	496	0.141	123	0.140	128	0.156	145	0.148	107	0.138
106	0.144	321	0.154	625	0.148	396	0.131	106	0.144	729	0.140	530	0.139	321	0.154	625	0.148	466	0.137
604	0.144	166	0.154	48	0.147	341	0.131	604	0.144	176	0.140	321	0.139	166	0.154	48	0.147	49	0.137
14	0.144	675	0.153	441	0.147	696	0.131	14	0.144	575	0.139	568	0.138	675	0.153	441	0.147	729	0.137
37	0.144	568	0.151	179	0.146	62	0.130	37	0.144	488	0.138	443	0.136	568	0.151	179	0.146	113	0.137
302	0.143	607	0.150	604	0.145	107	0.130	302	0.143	601	0.137	623	0.136	607	0.150	604	0.145	576	0.137
154	0.142	41	0.150	717	0.144	46	0.129	154	0.142	604	0.137	718	0.134	41	0.150	717	0.144	28	0.137
401	0.141	473	0.150	558	0.144	73	0.129	401	0.141	459	0.137	586	0.134	473	0.150	558	0.144	47	0.137
86	0.139	608	0.149	242	0.144	227	0.128	86	0.139	715	0.135	154	0.134	608	0.149	242	0.144	86	0.136
575	0.138	587	0.149	562	0.144	28	0.128	575	0.138	321	0.135	608	0.133	587	0.149	562	0.144	661	0.135
66	0.138	648	0.148	328	0.143	459	0.128	66	0.138	179	0.134	327	0.133	648	0.148	328	0.143	287	0.135
523	0.138	22	0.148	523	0.143	122	0.127	523	0.138	182	0.132	726	0.133	22	0.148	523	0.143	646	0.135
490	0.138	604	0.147	691	0.143	489	0.127	490	0.138	6	0.132	653	0.132	604	0.147	691	0.143	592	0.135
266	0.137	444	0.146	65	0.142	659	0.127	266	0.137	302	0.131	6	0.131	444	0.146	65	0.142	575	0.135
673	0.137	66	0.146	112	0.142	328	0.127	673	0.137	161	0.131	661	0.130	66	0.146	112	0.142	571	0.133
322	0.137	601	0.145	496	0.142	628	0.127	322	0.137	145	0.130	73	0.129	601	0.145	496	0.142	48	0.133
328	0.137	439	0.145	393	0.142	646	0.127	328	0.137	227	0.129	181	0.129	439	0.145	393	0.142	510	0.132
20	0.136	267	0.145	68	0.142	607	0.126	20	0.136	622	0.127	227	0.129	267	0.145	68	0.142	562	0.132
148	0.136	107	0.145	46	0.140	571	0.126	148	0.136	576	0.126	14	0.129	107	0.145	46	0.140	179	0.132
202	0.136	441	0.145	459	0.139	151	0.125	202	0.136	62	0.126	83	0.129	441	0.145	459	0.139	141	0.131
321	0.136	83	0.145	614	0.139	627	0.125	321	0.136	8	0.126	267	0.128	83	0.145	614	0.139	473	0.131
4	0.135	148	0.144	129	0.138	148	0.125	4	0.135	437	0.125	574	0.128	148	0.144	129	0.138	302	0.130
597	0.135	676	0.143	294	0.138	561	0.125	597	0.135	129	0.124	562	0.127	676	0.143	294	0.138	630	0.130
387	0.134	498	0.143	319	0.138	524	0.125	387	0.134	650	0.124	302	0.127	498	0.143	319	0.138	696	0.129
122	0.134	234	0.143	697	0.137	156	0.125	122	0.134	625	0.124	459	0.127	234	0.143	697	0.137	598	0.128
550	0.133	687	0.142	613	0.137	85	0.124	550	0.133	48	0.124	283	0.127	687	0.142	613	0.137	627	0.128
595	0.133	122	0.142	106	0.136	22	0.124	595	0.133	202	0.124	401	0.125	122	0.142	106	0.136	56	0.125
341	0.133	451	0.141	592	0.136	302	0.124	341	0.133	69	0.123	163	0.125	451	0.141	592	0.136	687	0.125
592	0.132	625	0.140	49	0.135	387	0.123	592	0.132	231	0.123	593	0.125	625	0.140	49	0.135	674	0.125
319	0.131	348	0.140	574	0.135	686	0.122	319	0.131	341	0.122	141	0.124	348	0.140	574	0.135	328	0.125
676	0.131	510	0.140	675	0.134	163	0.121	676	0.131	603	0.122	388	0.124	510	0.140	675	0.134	133	0.124
73	0.130	319	0.140	387	0.134	717	0.120	73	0.130	587	0.122	665	0.124	319	0.140	387	0.134	610	0.124
157	0.128	60	0.140	402	0.134	574	0.119	157	0.128	718	0.121	500	0.124	60	0.140	402	0.134	486	0.124
166	0.127	123	0.139	227	0.134	441	0.119	166	0.127	55	0.120	106	0.124	123	0.139	227	0.134	395	0.123
637	0.127	113	0.139	449	0.133	267	0.119	637	0.127	390	0.120	558	0.123	113	0.139	449	0.133	26	0.123

665	0.127	614	0.139	634	0.132	8	0.119	665	0.127	265	0.120	71	0.122	614	0.139	634	0.132	686	0.122
443	0.126	121	0.139	37	0.132	145	0.119	443	0.126	159	0.120	706	0.122	121	0.139	37	0.132	390	0.121
646	0.125	558	0.138	39	0.132	622	0.119	646	0.125	520	0.120	151	0.121	558	0.138	39	0.132	654	0.119
315	0.125	726	0.137	658	0.131	621	0.119	315	0.125	697	0.119	55	0.120	726	0.137	658	0.131	722	0.119
717	0.124	342	0.137	654	0.131	14	0.118	717	0.124	14	0.119	622	0.119	342	0.137	654	0.131	673	0.118
506	0.124	26	0.137	47	0.131	234	0.118	506	0.124	436	0.119	555	0.119	26	0.137	47	0.131	226	0.118
13	0.123	702	0.136	36	0.130	187	0.118	13	0.123	327	0.117	605	0.119	702	0.136	36	0.130	606	0.117
183	0.123	562	0.136	20	0.129	167	0.117	183	0.123	592	0.116	85	0.119	562	0.136	20	0.129	116	0.117
429	0.122	86	0.136	322	0.129	395	0.117	429	0.122	439	0.115	330	0.119	86	0.136	322	0.129	20	0.117
291	0.122	646	0.136	676	0.129	498	0.117	291	0.122	85	0.114	676	0.118	646	0.136	676	0.129	145	0.116
654	0.121	279	0.135	537	0.129	510	0.117	654	0.121	627	0.114	149	0.118	279	0.135	537	0.129	55	0.115
715	0.121	106	0.134	26	0.129	47	0.116	715	0.121	20	0.114	28	0.118	106	0.134	26	0.129	183	0.114
557	0.120	202	0.133	166	0.129	665	0.116	557	0.120	514	0.113	607	0.117	202	0.133	166	0.129	66	0.114
621	0.120	302	0.131	598	0.128	149	0.115	621	0.120	524	0.113	166	0.116	302	0.131	598	0.128	441	0.113
348	0.119	506	0.131	555	0.128	558	0.115	348	0.119	654	0.113	179	0.116	506	0.131	555	0.128	288	0.113
585	0.119	36	0.131	154	0.126	129	0.115	585	0.119	71	0.112	49	0.116	36	0.131	154	0.126	40	0.113
607	0.117	696	0.131	103	0.126	715	0.115	607	0.117	40	0.112	266	0.116	696	0.131	103	0.126	37	0.111
399	0.117	729	0.128	173	0.126	438	0.114	399	0.117	43	0.111	70	0.115	729	0.128	173	0.126	613	0.111
28	0.117	390	0.127	640	0.126	231	0.114	28	0.117	194	0.111	139	0.115	390	0.127	640	0.126	39	0.110
439	0.117	62	0.127	395	0.125	52	0.114	439	0.117	151	0.111	571	0.115	62	0.127	395	0.125	159	0.110
49	0.117	238	0.126	630	0.125	439	0.113	49	0.117	108	0.111	46	0.114	238	0.126	630	0.125	670	0.110
227	0.117	154	0.125	556	0.125	159	0.113	227	0.117	144	0.110	439	0.114	154	0.125	556	0.125	586	0.110
598	0.116	49	0.125	386	0.123	322	0.113	598	0.116	49	0.110	630	0.114	49	0.125	386	0.123	13	0.110
231	0.116	686	0.125	265	0.122	606	0.112	231	0.116	287	0.110	279	0.114	686	0.125	265	0.122	388	0.110
210	0.116	586	0.125	167	0.122	21	0.111	210	0.116	486	0.109	35	0.114	586	0.125	167	0.122	595	0.110
41	0.116	133	0.125	285	0.122	575	0.111	41	0.116	116	0.108	113	0.113	133	0.125	285	0.122	65	0.109
176	0.115	524	0.124	11	0.122	490	0.111	176	0.115	630	0.108	597	0.112	524	0.124	11	0.122	285	0.109
129	0.115	486	0.123	321	0.121	576	0.111	129	0.115	498	0.108	285	0.112	486	0.123	321	0.121	129	0.109
232	0.115	627	0.122	60	0.121	451	0.111	232	0.115	451	0.108	20	0.111	627	0.122	60	0.121	322	0.109
549	0.113	37	0.122	674	0.120	568	0.111	549	0.113	607	0.108	87	0.111	37	0.122	674	0.120	105	0.108
35	0.113	145	0.119	128	0.120	342	0.110	35	0.113	121	0.108	436	0.111	145	0.119	128	0.120	199	0.108
263	0.113	341	0.118	479	0.120	401	0.110	263	0.113	166	0.107	56	0.110	341	0.118	479	0.120	181	0.108
56	0.113	658	0.117	686	0.120	182	0.110	56	0.113	133	0.107	722	0.110	658	0.117	686	0.120	717	0.108
650	0.112	591	0.116	559	0.119	676	0.109	650	0.112	675	0.107	595	0.110	591	0.116	559	0.119	623	0.107
617	0.111	386	0.116	266	0.119	479	0.109	617	0.111	114	0.107	387	0.109	386	0.116	266	0.119	8	0.107
36	0.111	55	0.115	335	0.118	162	0.109	36	0.111	441	0.107	21	0.109	55	0.115	335	0.118	291	0.107
144	0.111	715	0.115	116	0.118	176	0.107	144	0.111	15	0.106	650	0.109	715	0.115	116	0.118	675	0.107
103	0.110	467	0.115	608	0.118	496	0.107	103	0.110	489	0.106	655	0.109	467	0.115	608	0.118	335	0.106
402	0.110	288	0.114	665	0.117	595	0.107	402	0.110	446	0.106	441	0.108	288	0.114	665	0.117	261	0.106
452	0.110	717	0.114	202	0.117	285	0.107	452	0.110	359	0.105	715	0.108	717	0.114	202	0.117	325	0.106
234	0.109	283	0.113	498	0.116	330	0.106	234	0.109	473	0.105	148	0.108	283	0.113	498	0.116	320	0.106
436	0.108	634	0.113	161	0.115	386	0.106	436	0.108	185	0.105	8	0.107	634	0.113	161	0.115	676	0.106
60	0.108	265	0.113	141	0.115	629	0.106	60	0.108	686	0.105	66	0.107	265	0.113	141	0.115	227	0.105
658	0.108	622	0.113	108	0.114	23	0.106	658	0.108	704	0.105	576	0.107	622	0.113	108	0.114	393	0.105
729	0.108	181	0.113	637	0.114	91	0.105	729	0.108	429	0.105	328	0.107	181	0.113	637	0.114	53	0.104
161	0.108	537	0.113	144	0.114	446	0.104	161	0.108	557	0.105	393	0.107	537	0.113	144	0.114	279	0.104
498	0.108	159	0.113	27	0.113	603	0.104	498	0.108	234	0.104	286	0.107	159	0.113	27	0.113	655	0.103
268	0.108	322	0.112	489	0.113	587	0.104	268	0.108	682	0.104	431	0.106	322	0.112	489	0.113	148	0.103
562	0.107	151	0.112	550	0.113	436	0.104	562	0.107	605	0.104	40	0.106	151	0.112	550	0.113	591	0.102
215	0.107	268	0.111	605	0.112	608	0.104	215	0.107	719	0.104	103	0.106	268	0.111	605	0.112	158	0.102
471	0.107	287	0.111	283	0.112	174	0.103	471	0.107	628	0.104	60	0.106	287	0.111	283	0.112	223	0.102
271	0.106	396	0.111	107	0.112	506	0.103	271	0.106	267	0.104	32	0.106	396	0.111	107	0.112	659	0.102
44	0.106	503	0.111	444	0.112	70	0.103	44	0.106	655	0.104	115	0.106	503	0.111	444	0.112	267	0.102

459	0.106	613	0.110	83	0.111	20	0.103	459	0.106	113	0.104	156	0.106	613	0.110	83	0.111	535	0.102
301	0.106	452	0.110	627	0.111	336	0.102	301	0.106	35	0.103	673	0.105	452	0.110	627	0.111	557	0.101
390	0.105	134	0.110	622	0.111	488	0.102	390	0.105	60	0.103	159	0.104	134	0.110	622	0.111	234	0.101
586	0.104	379	0.110	43	0.111	327	0.102	586	0.104	593	0.103	400	0.104	379	0.110	43	0.111	550	0.101
39	0.104	606	0.109	151	0.111	49	0.102	39	0.104	65	0.103	128	0.104	606	0.109	151	0.111		
83	0.102	628	0.109	181	0.110	402	0.101	83	0.102	156	0.103	47	0.104	628	0.109	181	0.110		
687	0.102	11	0.109	389	0.110	591	0.101	687	0.102	360	0.103	575	0.103	11	0.109	389	0.110		
411	0.102	225	0.109	327	0.109	55	0.101	411	0.102	597	0.103	226	0.103	225	0.109	327	0.109		
185	0.102	556	0.109	397	0.109	399	0.101	185	0.102	10	0.102	614	0.102	556	0.109	397	0.109		
267	0.101	555	0.109	21	0.109	674	0.100	267	0.101	319	0.102	563	0.102	555	0.109	21	0.109		
634	0.101	112	0.108	197	0.108			634	0.101	162	0.102	265	0.101	112	0.108	197	0.108		
614	0.101	561	0.107	73	0.108			614	0.101	268	0.102	185	0.101	561	0.107	73	0.108		
85	0.101	194	0.107	74	0.108			85	0.101	388	0.101	191	0.100	194	0.107	74	0.108		
46	0.100	64	0.107	288	0.107			46	0.100	558	0.101			64	0.107	288	0.107		
181	0.100	660	0.106	419	0.107			181	0.100	550	0.101			660	0.106	419	0.107		
		617	0.106	486	0.107									617	0.106	486	0.107		
		286	0.106	55	0.107									286	0.106	55	0.107		
		547	0.106	148	0.106									547	0.106	148	0.106		
		70	0.106	563	0.106									70	0.106	563	0.106		
		409	0.106	187	0.106									409	0.106	187	0.106		
		592	0.105	62	0.106									592	0.105	62	0.106		
		665	0.105	175	0.105									665	0.105	175	0.105		
		639	0.105	425	0.105									639	0.105	425	0.105		
		559	0.105	536	0.105									559	0.105	536	0.105		
		697	0.105	400	0.105									697	0.105	400	0.105		
		56	0.105	575	0.105									56	0.105	575	0.105		
		654	0.105	718	0.105									654	0.105	718	0.105		
		483	0.105	176	0.105									483	0.105	176	0.105		
		48	0.105	619	0.105									48	0.105	619	0.105		
		593	0.105	524	0.104									593	0.105	524	0.104		
		323	0.104	586	0.104									323	0.104	586	0.104		
		175	0.104	549	0.104									175	0.104	549	0.104		
		550	0.104	139	0.104									550	0.104	139	0.104		
		74	0.104	593	0.104									74	0.104	593	0.104		
		131	0.104	399	0.103									131	0.104	399	0.103		
		87	0.103	274	0.103									87	0.103	274	0.103		
		285	0.103	452	0.103									285	0.103	452	0.103		
		183	0.103	199	0.103									183	0.103	199	0.103		
		387	0.103	234	0.103									387	0.103	234	0.103		
		653	0.103	568	0.103									653	0.103	568	0.103		
		619	0.102	467	0.103									619	0.102	467	0.103		
		224	0.101	126	0.101									224	0.101	126	0.101		
		69	0.101	225	0.101									69	0.101	225	0.101		
		560	0.101	528	0.100									560	0.101	528	0.100		
		200	0.100	180	0.100									200	0.100	180	0.100		
				597	0.100											597	0.100		
